# Impact of combining indoor residual spraying and long-lasting insecticidal nets on *Anopheles arabiensis* in Ethiopia: results from a cluster randomized controlled trial

**DOI:** 10.1186/s12936-019-2811-1

**Published:** 2019-05-24

**Authors:** Oljira Kenea, Meshesha Balkew, Habte Tekie, Wakgari Deressa, Eskindir Loha, Bernt Lindtjørn, Hans J. Overgaard

**Affiliations:** 1 Department of Zoological Sciences, Addis Ababa University, Addis Ababa, Ethiopia; 2 Department of Biology, Wollega University, Nekemte, Ethiopia; 3 Akililu Lemma Institute of Pathobiology, Addis Ababa University, Addis Ababa, Ethiopia; 4 Department of Preventive Medicine, School of Public Health, College of Health Sciences, Addis Ababa University, Addis Ababa, Ethiopia; 5 School of Public and Environmental Health, Hawassa University, Hawassa, Ethiopia; 6 Centre for International Health, University of Bergen, Bergen, Norway; 7 Faculty of Science and Technology, Norwegian University of Life Sciences, Ås, Norway

**Keywords:** *Anopheles arabiensis*, Ethiopia, Long-lasting insecticidal nets, Malaria

## Abstract

**Background:**

Indoor residual house spraying (IRS) and long-lasting insecticidal nets (LLINs) are the key front-line malaria vector interventions against *Anopheles arabiensis*, the sole primary malaria vector in Ethiopia. Universal coverage of both interventions has been promoted and there is a growing demand in combinations of interventions for malaria control and elimination. This study compared the impact on entomological outcomes of combining IRS and LLINs with either intervention alone in Adami Tullu district, south-central Ethiopia. The epidemiological outcomes were recently published on a separate paper.

**Methods:**

This factorial, cluster-randomized, controlled trial randomized villages to four study arms: IRS + LLIN, IRS, LLIN, and control. LLINs (PermaNet 2.0) were provided free of charge. IRS with propoxur was applied before the main malaria transmission season in 2014, 2015 and 2016. Adult mosquitoes were collected in randomly selected villages in each arm using CDC light trap catch (LTC) set close to a sleeping person, pyrethrum spray catch (PSC), and artificial pit shelter (PIT), for measuring mosquito host-seeking density (HSD), indoor resting density (IRD), and outdoor resting density (ORD), respectively. Human landing catch (HLC) was performed in a sub-set of villages to monitor *An. arabiensis* human biting rates (HBR). Mean vector densities and HBR were compared among study arms using incidence rate ratio (IRR) calculated by negative binomial regression.

**Results:**

There were no significant differences in mean densities (HSD, IRD, ORD) and HBR of *An. arabiensis* between the IRS + LLIN arm and the IRS arm (p > 0.05). However, mean HSD, IRD, ORD, and HBR were significantly lower in the IRS + LLIN arm than in the LLIN alone arm (p < 0.05). All *An. arabiensis* tested for malaria infection were negative for *Plasmodium* species. For this reason, the entomological inoculation rate could not be determined.

**Conclusions:**

The IRS + LLIN were as effective as IRS alone in reducing densities and HBR of *An. arabiensis.* However, the effectiveness of the two interventions combined was higher than LLINs alone in reducing densities and HBR of the vector. Added impact of the combination intervention against malaria infectivity rates of *An. arabiensis* compared to either intervention alone remains unknown and warrants further research.

*Trial registration* PACTR201411000882128. Registered 8 September 2014, https://trialsjournal.biomedcentral.com/articles/10.1186/s13063-016-1154-2

## Background

Malaria remains a major health problem in Ethiopia where only 25% of the population live in areas that are free from malaria [1]. It is among the ten top leading causes of morbidity and mortality in children under 5 years old [2]. Malaria transmission is seasonal and epidemic in Ethiopia, mainly due to altitudinal and climatic variations [3]. High malaria transmission intensity occurs as *Anopheles arabiensis* populations expand during the wet seasons. Malaria transmission peaks from September to December coinciding with the major rainy season. A minor transmission season also occurs in April–May [3]. *Anopheles arabiensis* is the sole primary malaria vector in Ethiopia [4]. It transmits *Plasmodium falciparum* and *Plasmodium vivax*, the dominant malaria parasites, which account for around 60 and 40% of the all malaria cases in the country, respectively [1].

Indoor residual house spraying **(**IRS) and long-lasting insecticidal nets (LLINs) are the key front-line life-saving malaria vector interventions against *An. arabiensis* in Ethiopia. In malaria vector interventions either of IRS and LLINs can be applied singly or in an integrated manner [5]. IRS kills mosquitoes or reduces longevity when they rest on insecticide-sprayed surfaces inside houses, before and after feeding on occupants. LLINs reduce malaria parasite transmission mainly by killing or blocking mosquitoes that attempt to feed on humans under net [6].

In Ethiopia, IRS and LLINs are scaled-up and intensively implemented in combination or separately for malaria control interventions, primarily targeting *An. arabiensis* [5]. However, there is contradictory evidence whether or not the combination intervention is better than implemented separately [6]. Cluster randomized trials provide the best evidence for the effectiveness of such interventions [6]. Trials have been completed in Benin, The Gambia and Tanzania to investigate whether or not combination provides added protection compared to insecticide-treated nets (ITNs) alone. The outcome measures of the Benin trial were incidence density rates of *P. falciparum* clinical malaria in children younger than 6 years, entomological inoculation rates (EIR) and human biting rates (HBR) of the primary malaria vector *Anopheles gambiae* sensu stricto (s.s.) [7]. However, the trial results showed that none of the outcome measures was significantly reduced in IRS and LLIN combination as compared to LLIN alone indicating that there was no evidence of added protection from the combination intervention [7]. The Gambia trial compared the incidence of clinical malaria assessed by passive case detection in children 0.5–14 years old, and density and EIR of *An. gambiae* sensu lato (s.l.) collected per light trap per night in LLINs in combination with IRS versus LLINs alone. Also in this trial there were no significant differences between the study arms, indicating that IRS offered no increased protection compared to the use of LLINs alone [8].

The Tanzanian trial compared *P. falciparum* prevalence rate in children 0.5–14 years old, and density and EIR of *An. gambiae* s.s. between the combined intervention of ITN and IRS versus ITN alone. This trial provided the first conclusive evidence that combining IRS and ITNs produces major reductions in malaria infection prevalence and in *Anopheles* density and EIR compared to ITN alone [9, 10]. These trials assessed the effects on *An. gambiae,* but trials targeting *An. arabiensis* are limited.

The previous trials compared epidemiological outcomes in communities receiving IRS + LLIN versus those receiving LLINs alone, but no trials have so far compared the standardized IRS + LLIN versus IRS alone. Furthermore, these trials did not have controls in the same way as the current trial. Because evidence is needed to determine the effectiveness of combining IRS and LLINs in any transmission setting, WHO recommends that countries that are already using both interventions in combination should undertake an evaluation of the effectiveness of the combination versus either LLINs or IRS alone [6].

Entomological outcomes of IRS and LLIN combination intervention trials that target *An. arabiensis* is lacking in Ethiopia. IRS and LLIN combined intervention trial results elsewhere in Africa on *An. gambiae* s.l. described earlier [7–10] are not necessarily relevant for *An. arabiensis* because of locally variable environmental factors and unique bionomics of *An. arabiensis* and local anthropological factors. Therefore, this study assessed the impact of combined and separate interventions on vector density and HBR of *An. arabiensis* in Adami Tullu district, south-central Ethiopia. This study aimed to answer the following research questions: Does the combined use of IRS and LLINs significantly reduce vector density as compared to their separate use? Does IRS and LLINs co-application significantly affect *An. arabiensis* biting rates versus IRS or LLINs alone? The main hypothesis of this study is that the combined use of IRS and LLINs will significantly reduce vector density and HBR as compared to either their separate use or the control group. The epidemiological results of the trial were published in a separate paper [11]. In this paper, the effect of the interventions on the vector densities and HBR is reported.

## Methods

### Study area

**Fig. 1:**
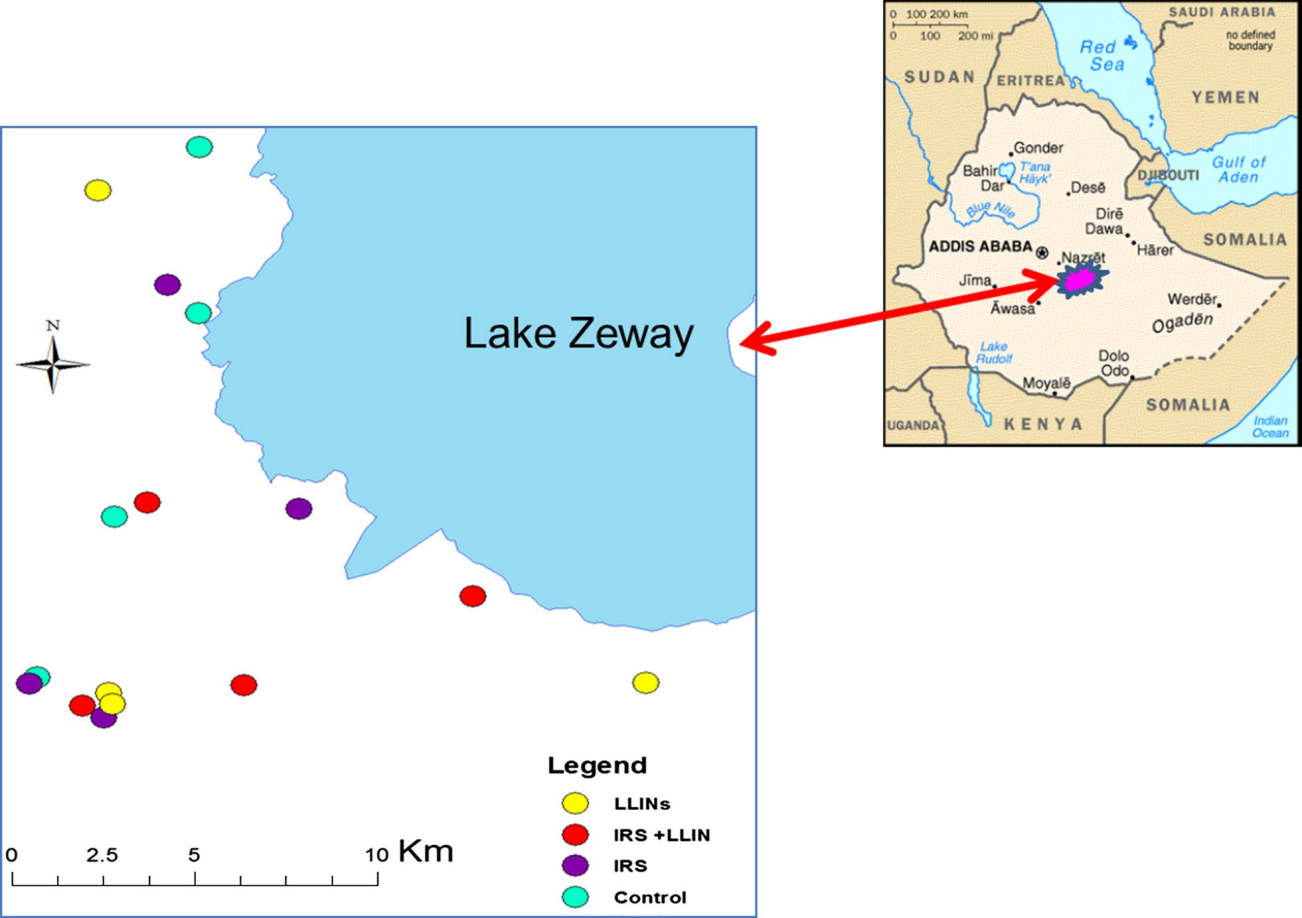
Distribution of village clusters selected for entomological sampling in the study setting, Adami Tullu, 2014–2016

The study area is located at 7°56′ N 38°42′ E; 1640 m above sea level about 160 km south of Addis Ababa on the highway connecting Addis Ababa with Nairobi. The area has been described in detail in the published trial protocol [12] and elsewhere [13–15]. Briefly, the study was carried out in villages in 13 *kebeles* located within a 5-km distance from Lake Zeway and Bulbula River in Adami Tullu district, south-central Ethiopia (Fig. 1). This area has many breeding sites and more mosquitoes than areas farther away from the lake and the river. A village contains about 35 households and is defined as a geographical division of a *kebele*. Most of the population in the district live in rural areas in houses made of mud or cement walls and thatched or corrugated iron roofs. Local residents primarily depend on farming, livestock rearing, and fishing for subsistence from Lake Zeway. Data collections were carried out during the 2014, 2015 and 2016 major malaria transmission seasons, which usually are from September to November.

### Study design and randomization

This is a 2 × 2 factorial, cluster-randomized, controlled trial (CRT) consisting of IRS, LLIN, IRS + LLIN, and a control. The unit of randomization for the intervention was village (cluster). Each arm contained 44 clusters for epidemiological outcomes (results reported elsewhere), but only four clusters per arm were included for the entomological outcomes reported here. The four clusters per arm were randomly selected from the 44. Randomization was done by a computer-generated list using SPSS software.

### Intervention

All households in the IRS + LLIN and LLIN arms of the trial received new LLINs free of charge provided by the project. The LLINs used for this trial were PermaNet 2.0 rectangular 100 denier, purchased in June 2014 from Vestergaard Frandsen Group SA (Vestergaard Frandsen, Lausanne, Switzerland). PermaNet 2.0 is a WHO approved factory-treated mosquito net manufactured with deltamethrin at 55 mg active ingredient per sq m, that is expected to retain its biological efficacy for a minimum of 20 standard WHO washes [12]. The life span of LLINs is about 3 years under field conditions [16], long enough to cover the study period. The target households received light-blue family size (160 cm width × 180 cm length × 150 cm height) models according to the number of LLINs recommended based on family size. The national malaria guidelines recommends one net for a family of 1–2 persons, two nets for a family of 3–5 persons, three nets for a family of 6–7 persons and four nets for a family of 8 and above people [12, 17].

Propoxur was used for the IRS with one spray round per year prior to the peak transmission season. Propoxur is an isopropoxy-phenyl methyl carbamate highly effective against mosquito vectors for 3–6 months at the dosage of 2 g/sq m in the form of a water-dispersible powder [12]. The residual activity of propoxur is sufficient to cover the main malaria season. The insecticide was purchased in 2014 from the state-owned Adami Tullu Pesticide Processing Share Company located in the study district. Propoxur 50% contains 2 g of active ingredient and is packaged in 400 g sachets. Two sachets (800 g) were mixed in 8 L of water. The interior walls and ceilings of each dwelling were sprayed with propoxur at 2 g/sq m using an 8 L Hudson X-pertsprayer (HD Hudson Manufacturing Company, Chicago, IL USA) following the national spraying operation guidelines [12, 17].

The control arm received routine practice of malaria prevention by the District Health Office (DHO) as described in the previous protocol [12]. The control households would receive new LLINs and IRS spraying when the DHO found it appropriate, but during the study period, no communities in the study area received such additional interventions. All people living in the area were offered malaria diagnosis and treatment, if needed, when presenting at a health institution as per the protocol reported earlier [12].

Due to the nature of the interventions, blinding of the study participants was not possible. Mosquito collector bias was reduced using automated standard mosquito traps.

### Mosquito collections and sporozoite detection

Malaria vectors were collected in randomly selected houses using light trap catches (LTC), pyrethrum spray catches (PSC), and artificial outdoor pit shelters (PIT). LTC and PIT were placed in one house per cluster. PSC was performed in four houses per cluster. LTC, PSC and PIT were used to monitor the impact of the interventions on *An. arabiensis* host-seeking density (HSD), indoor resting density (IRD) and outdoor resting density (ORD), respectively. In addition, HLC was performed indoors and outdoors in one house in one cluster per study arm to monitor the impact the interventions on *An. arabiensis* HBR. Indoor HSD was estimated by LTC than HLC to reduce mosquito collector bias using automated standard mosquito traps. The LTC, PSC and PIT were done during three malaria seasons in 2014, 2015 and 2016 for three alternative nights/days per week, whereas the HLC was only done during two malaria seasons in 2015 and 2016 for two alternative nights per week. The power of the entomological study was calculated using methods for cluster-randomized trials. Four households per cluster in four clusters per arm were followed up for 36 weeks achieving 80% power to detect a 25% reduction in mosquito density in the LLIN + IRS arm compared to LLIN arm using a two-sided 5% significance level.

Direct sporozoite ELISA was carried out for determination of *Plasmodium falciparum* and *Plasmodium vivax* sporozoites rates. Overall, 574 (61.8%) of all mosquito specimens obtained by all collection methods were tested for the sporozoites using methods described by Beier et al. [18].

### Ethical considerations

Ethical approval was obtained from the Institutional Review Board of the College of Health Sciences at Addis Ababa University, Ministry of Science and Technology, Ethiopia (Ref: 3.10/446/06), and the Regional Committee for Medical and Health Research Ethics, Norway (Ref: 2013/986/REK Vest). The protocol for the trial was registered at PACTR201411000882128. Detailed ethical considerations have been described in the published protocol [12] and in a recent publication from the same project [15].

Verbal and written informed consent, using local language, was obtained beforehand from mosquito collectors, who were all older than 18 years, describing the potential risks and benefits of the study. Verbal and written informed consent was also obtained from house owners. Mosquito collectors were trained how to collect mosquitoes without being bitten. To help minimize risk, mosquito collectors were provided with an appropriate prophylactic drug (Malarone). There were no reports on malarone resistant *Plasmodium* parasites in Ethiopia. The project provided blood examination and treatment of malaria free of charge for any study participant or householder who fell ill or wished to check himself. The project follows the examination and treatment guidelines as described in the study protocol [12].

### Data analysis

Mean mosquito densities obtained by different sampling methods were compared among the study arms. Indoor host-seeking *An. arabiensis* density (HSD) was assessed by indoor LTC and calculated as the total number of *An. arabiensis* collected divided by the total number of light trap collection nights (mosquitoes/trap/night). IRD was assessed by PSC and expressed as the total number of *An. arabiensis* divided by the number of houses and collection days (mosquitoes/house/day). ORD was assessed by PIT and calculated as the total number of *An. arabiensis* divided by the number of pits and collection days (mosquitoes/pit/day).

Mean mosquito HBR were obtained by LTC and HLC and compared among study arms. Indoor HBR was estimated by LTC expressed as the total number of mosquitoes caught in the light trap divided by a conversion factor of 0.35 for *An. arabiensis,* representing species-specific relative efficiency to account for the lower efficiency of LTC relative to HLC [15]. The HBR for indoor LTC was not adjusted for the number of household inhabitants because it was considered proportionally representative of true adult exposure [19]. For mosquito collections by HLC, the real HBR was directly calculated as the mean number of bites received per person per night of collection (b/p/n) [20].

Mean densities and HBR of *An. arabiensis* collected by each mosquito sampling method was compared among study arms using negative binomial regression in generalized linear models (GLM). The impact of interventions on vector indices (vector parameters) was therefore estimated by exponentiation of the negative binomial regression coefficient, i.e., incidence rate ratio (IRR) at *p* value < 0.05 significance level. Two analyses per collection method were done: (1) comparing the three intervention arms against the control arm; and, (2) comparing the single intervention arms only against the IRS + LLIN arm, i.e., only comparing the three interventions against each other and excluding the control arm. All statistical analyses were done using SPSS version 20.0.

## Results

### *Anopheles arabiensis* abundance

**Fig. 2:**
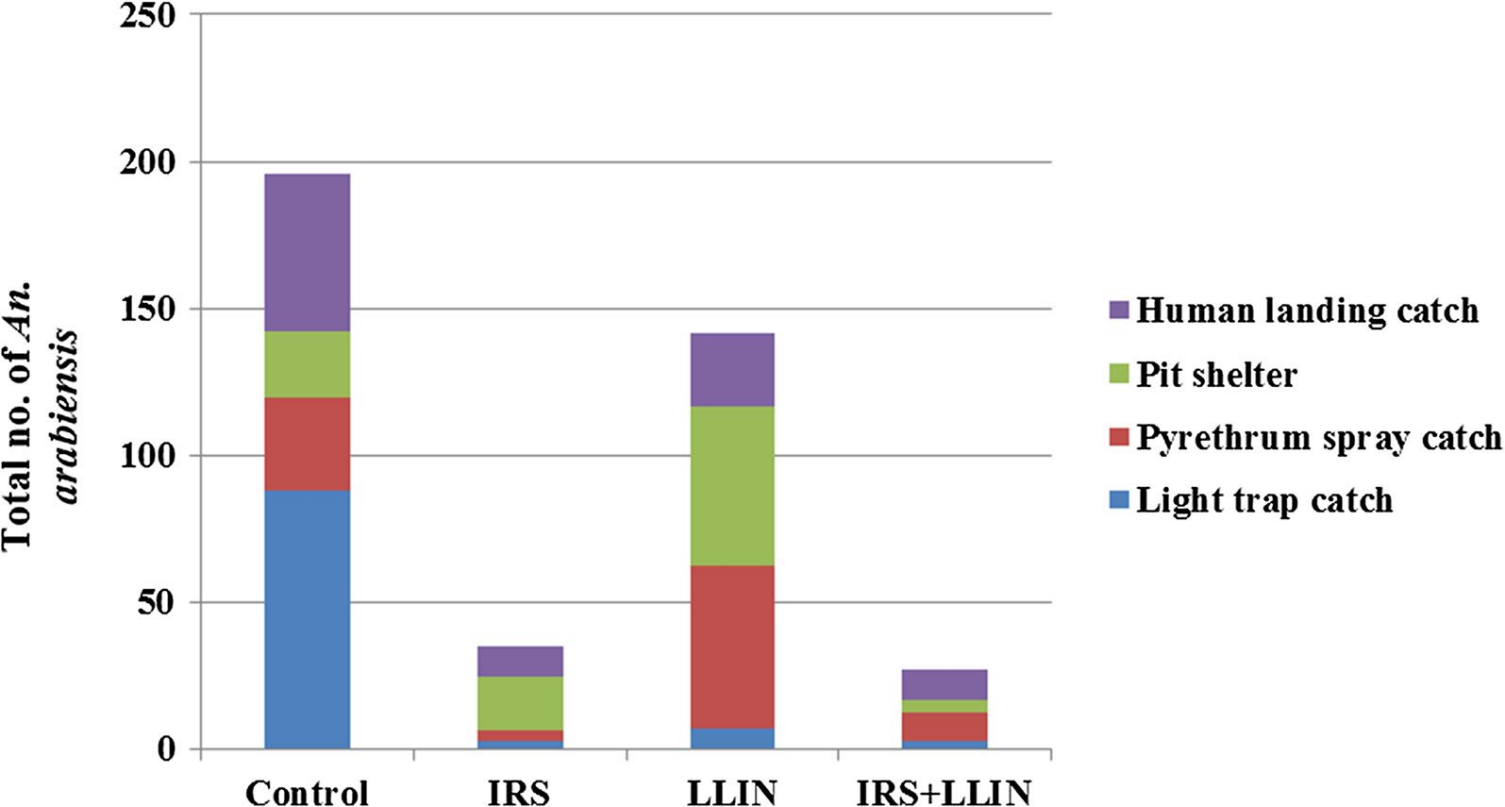
Number of *Anopheles arabiensis* collected by light trap catches (LTC), pyrethrum spray catches (PSC), pit shelter (PIT) and human landing catches (HLC) by study arms in Adami Tullu, Ethiopia

Altogether 929 female *An. arabiensis* were collected, being most abundant in the control arm (56.9%) followed by the LLIN arm (25.6%), and least abundant in the IRS (9.0%) and the IRS + LLIN (8.4%) arms (Fig. 2). The LTC collected most mosquitoes in the control arm (87.7%) and least in the IRS (2.8%) and IRS + LLIN (2.4%) arms. The PSC collected most mosquitoes in the LLIN arm (55.3%) and least in the IRS arm (3.5%). The PIT collected most in the LLIN arm (54.5%), followed by the control arm (22.7%), the IRS (18.2%), and combined intervention arm (4.5%). Using HLC, the vector was most frequently collected in the control arm (53.5%) and least from the combination arm (10.3).

### Comparison of *Anopheles arabiensis* densities among the study arms

#### Indoor host-seeking density (HSD)

The mean indoor HSD of *An. arabiensis* assessed by indoor LTC was 1.11, 0.03, 0.09 and 0.03 mosquitoes/trap/night in the control, IRS, LLINs, and IRS + LLINs arms, respectively (Table 1a, b). The mean HSD of *An. arabiensis* in the control arm was significantly higher compared to each of the intervention arms (p < 0.001). Nevertheless, among the intervention arms mean *An. arabiensis* HSD in the LLINs alone was higher, but not significantly so, compared to the IRS + LLINs (p = 0.076). There were no significant differences in vector HSD between IRS + LLINs and IRS (p = 1.000).

**Table 1:** Indoor host-seeking density using light trap catches and incidence rate ratios in intervention and control groups of *Anopheles arabiensis* in Adami Tullu, Ethiopia

Study arms	Person-night catch	Mean density (95% CI)	IRR (95% CI)	P value
a) Control arm is the reference group				
Control	592	1.11 (0.89–1.38)		
IRS	592	0.03 (0.01–0.08)	0.031 (0.012–0.076)	< 0.001
LLIN	592	0.09 (0.05–0.15)	0.096 (0.060–0.153)	< 0.001
IRS + LLIN	592	0.03 (0.01–0.08)	0.031 (0.012–0.076)	< 0.001
b) IRS + LLIN arm is the reference group				
IRS + LLIN	444	0.03 (0.01–0.08)		
IRS	444	0.03 (0.01–0.08)	1.424 (0.284–3.525)	1.000
LLIN	444	0.09 (0.05–0.15)	2.601 (0.903–7.478)	0.076

*IRS* indoor residual spray, *LLIN* long-lasting insecticidal nets, *LTC* light trap catch

#### Indoor resting density (IRD)

The mean IRD of *An. arabiensis* assessed by PSC was 0.19, 0.02, 0.34 and 0.06 mosquitoes/house/day in the control, IRS, LLIN and IRS + LLIN arms, respectively (Table 2). Compared to the control arm, the mean IRD was significantly lower in the IRS and the combination arms, respectively (p < 0.05), but the LLIN arm had a higher density (p < 0.05). Comparing the intervention arms only, there was no significant difference between the IRS + LLIN and IRS arms, but the LLIN arm had a higher density than the IRS + LLIN arm (Table 2b).

**Table 2:** Indoor resting density using pyrethrum spray catches and incidence rate ratios in intervention and control groups of *Anopheles arabiensis* in Adami Tullu, Ethiopia

Study arms	Person-night catch	Mean density (95% CI)	IRR (95% CI)	P value
a) Control arm is the reference group				
Control	560	0.19 (0.13–0.29)		
IRS	560	0.02 (0.01–0.07)	0.111 (0.033–0.374)	< 0.001
LLIN	560	0.34 (0.24–0.47)	1.740 (1.026–2.951)	0.040
IRS + LLIN	560	0.06 (0.03–0.12)	0.296 (0.130–0.675)	0.004
b) IRS + LLIN arm is the reference group				
IRS + LLIN	420	0.06 (0.03–0.12)		
IRS	420	0.02 (0.01–0.07)	0.375 (0.097–1.443)	0.154
LLIN	420	0.34 (0.24–0.47)	5.376 (2.406–12.013)	< 0.001

*IRS* indoor residual spray, *LLIN* long-lasting insecticidal nets, *PSC* pyrethrum spray catches

#### Outdoor resting density (ORD)

The mean ORD of *An. arabiensis* collected by PIT was 0.18, 0.14, 0.43 and 0.04 mosquitoes/pit/day in the control, IRS, LLIN and IRS + LLIN arms, respectively (Table 3a, b). There were no significant difference in the mean ORD between the control and the IRS arm (p > 0.05). However, mean ORD in the control arm was significantly higher compared to the combination arm (p < 0.05) but was significantly lower than that in the LLINs arm (p < 0.05). Comparing the intervention arms only, there was no significant difference between the IRS + LLIN and IRS arms, but the LLIN arm had a higher ORD than the IRS + LLIN arm (Table 3b).

**Table 3:** Outdoor resting density using artificial pit shelter and incidence rate ratios in intervention and control groups of *Anopheles arabiensis* in Adami Tullu, Ethiopia

Mosquito collection method and study arms	Collection nights	Mean density (95% CI)	IRR (95% CI)	p-value
a) Control arm is the reference group				
Control	224	0.18 (0.09–0.35)		
IRS	224	0.14 (0.07–0.30)	0.800 (0.294–2.177)	0.662
LLIN	224	0.43 (0.27–1.69)	2.398 (1.051–5.479)	0.038
IRS + LLIN	224	0.04 (0.01–0.15)	0.200 (0.042–0.954)	0.044
b) IRS + LLIN arm is the reference group				
IRS + LLIN	168	0.04 (0.01–0.15)		
IRS	168	0.14 (0.07–0.30)	3.998 (0.813–19.668)	0.088
LLIN	168	0.43 (0.27–1.69)	12.001 (2.707–53.196)	< 0.001

*IRS* indoor residual spray, *LLIN* long-lasting insecticidal nets, *PIT* outdoor artificial pit shelter

### Human biting rate (HBR) based on LTC

The mean HBR of *An. arabiensis,* as estimated using a conversion factor based on indoor LTC, was significantly higher in the control arm compared to each of intervention arms (Table 4a). However, the HBR in the IRS + LLIN arm was significantly lower than in the LLINs arm, but was not different from the IRS arm (Table 4b).

**Table 4:** Indoor human biting rates based on light trap catches and a conversion factor and incidence rate ratios in intervention and control groups of *Anopheles arabiensis* in the Adami Tullu, Ethiopia

Mosquito collection method and study arms	Collection nights	Mean density (95% CI)	IRR (95% CI)	p-value
a) Control is the reference group				
Control	592	3.15 (2.62–3.79)		
IRS	592	0.09 (0.05–0.15)	0.028 (0.015–0.051)	< 0.001
LLIN	592	0.26 (0.19–0.37)	0.084 (0.056–0.125)	< 0.001
IRS + LLIN	592	0.10 (0.06–0.17)	0.032 (0.018–0.056)	< 0.001
b) IRS + LLIN is the reference group				
IRS + LLIN	444	0.10 (0.06–0.17)		
IRS	444	0.09 (0.05–0.15)	0.867 (0.398–1.885)	0.718
LLIN	444	0.26 (0.19–0.37)	2.601 (1.374–4.918)	0.003

*IRS* indoor residual spray, *LLIN* long-lasting insecticidal nets, *LTC* light trap catches

### Human biting rate (HBR) based on HLC indoors

The mean HBR of *An. arabiensis* estimated by indoor HLC was significantly higher in the control arm than in the intervention arms (Table 5a). Among the intervention arms, the LLIN arm had the highest indoor HBR (Table 5b). However, there was no significant difference in mean HBR of *An. arabiensis* between the IRS and the IRS + LLIN arms indoors (p > 0.05).

**Table 5:** Indoor human biting rates using human landing catches and incidence rate ratios in intervention and control groups of *Anopheles arabiensis* in the Adami Tullu, Ethiopia

Study arms	Person-night catch	Mean biting density (95% CI)	IRR (95% CI)	p-value
a) Control is the reference group				
Control	76	9.89 (6.17–15.86)		
IRS	76	1.68 (0.95–2.97)	0.170 (0.081–0.356)	< 0.001
LLIN	76	3.95 (2.39–6.53)	0.399 (0.200–0.795)	0.009
IRS + LLIN	76	1.63 (0.92–2.89)	0.165 (0.078–0.346)	< 0.001
b) IRS + LLIN is the reference group				
IRS + LLIN	57	1.63 (0.92–2.89)		
IRS	57	1.68 (0.95–2.97)	1.032 (0.461–2.309)	0.938
LLIN	57	3.95 (2.39–6.53)	2.420 (1.129–5.181)	0.023

*IRS* indoor residual spray, *LLIN* long-lasting insecticidal nets, *HLC* human landing catches

### Human biting rate (HBR) based on HLC outdoors

The mean outdoor HBR of *An. arabiensis* estimated by outdoor HLC was significantly higher in the control arm compared to the intervention arms (Table 6a). Among the intervention arms, the LLIN arm had a significantly higher HBR than the combination arm (Table 6b). However, there was no significant difference in mean outdoor HBR between the IRS and the combination arm (p > 0.05).

**Table 6:** Outdoor human biting rates using human landing catches and incidence rate ratios in intervention and control groups of *Anopheles arabiensis* in the Adami Tullu, Ethiopia

Study arms	Person-night catch	Mean biting density (95% CI)	IRR (95% CI)	p-value
a) Control is the reference group				
Control	76	7.37 (4.56–11.90)		
IRS	76	1.89 (1.09–3.30)	0.257 (0.123–0.536)	< 0.001
LLIN	76	4.16 (2.52–6.86)	0.564 (0.282–1.128)	0.106
IRS + LLIN	76	1.68 (0.95–2.97)	0.228 (0.108–0.480)	< 0.001
b) IRS + LLIN is the reference group				
IRS + LLIN	57	1.68 (0.95–2.97)		
IRS	57	1.89 (1.09–3.30)	1.125 (0.508–2.489)	0.771
LLIN	57	4.16 (2.52–6.86)	2.469 (1.158–5.265)	0.019

*IRS* indoor residual spray, *LLIN* long-lasting insecticidal nets, *HLC* human landing catches

### Sporozoite rate and entomological inoculation rates

Altogether 574 (61.8%) *An. arabiensis* collected from all study arms were tested for the presence of *P. falciparum* and *P. vivax*. However none was found positive. For this reason, the EIR, which is the product of HBR and the sporozoite rate, could not be determined in this study.

## Discussion

The ultimate aim of this study was to examine the impact of IRS and LLIN individual versus combined interventions on *An. arabiensis* density, HBR and infectivity. Results showed that mean indoor densities and HBR of *An. arabiensis* were significantly lower in arms that were exposed to any of the interventions (IRS + LLIN, IRS, LLIN) compared to the control, or unexposed, group except mean IRD of the vector in the LLIN arm. These significant reductions implicate that the interventions were effective. This would be expected because IRS and LLINs applied either individually or jointly kill and/or repel mosquitoes when they attempt to feed and rest indoors, so that vector survival and population densities are reduced in intervention arms. These findings are consistent with several studies, which support that IRS and LLINs suppress both density and HBR of malaria vectors [21–24].

Unexpectedly, IRD of *An. arabiensis* was higher in the LLIN arm compared to the control arm. Several possible explanations are possible for this result. Since LLINs prevent blood feeding on an occupant as a chemical and/or physical barrier [25], the higher mean IRD in the LLIN arm could be because unfed mosquitoes are waiting indoors for opportunities to feed and outdoor-fed mosquitoes may rest indoors. In the control arm, to the contrary, indoor resting mosquitoes are expected to be mainly blood fed and gravid mosquitoes due to the higher access to blood meal sources. If this is the case, the LLIN insecticide does not seem to be effective enough to repel or kill indoor resting mosquitoes. Another possibility is that the LLIN reduces the potential area in the room where mosquitoes can, or prefer, to rest and thereby become concentrated in locations, which are more exposed to the pyrethrum spray compared to the control. Furthermore, LLINs are proven and effective; however impact also depends on the existence of a strong ‘net culture’ in the community. For example, proper use and care of nets is a key behaviour change that must take place if LLIN interventions are to be as effective as IRS [26]. A parallel community level assessment of LLIN coverage and use in the study area showed a low LLIN ownership after 110 weeks and a low LLIN use during 121 weeks of follow-up, despite 100% LLIN coverage at baseline [27].

Comparing the intervention arms only, mean indoor densities and HBR of *An. arabiensis* were significantly higher in the LLIN arm compared to the IRS + LLIN arm but they were similar in the IRS + LLIN versus the IRS arms. These results could be attributed to the potential basic differences in operational applications and efficacy between IRS and LLINs. At household level, IRS was applied to all potential mosquito resting places in human dwellings unlike LLINs which were positioned at human sleeping spaces (often limited to bed rooms) [12]. Therefore, the relatively larger area-wide coverage of IRS in the arms having an IRS intervention might have equally suppressed overall mosquito populations compared to the LLIN alone arm. Lack of convenient space to hang more than one net were usually reported as a key challenge that reduces proper utilization of LLINs in rural household settings [28, 29] and this challenge may contribute to the higher densities and HBR of the vector in the LLIN arm as well. Householders in the IRS + LLIN arm might stop using LLINs, potentially feeling sufficiently protected by the IRS and instead using their LLINs for unintended purposes, as was observed during the study period [30], coupled with the low coverage and use of LLINs explained earlier [27].

Furthermore, the higher indoor densities and HBR of the vector observed in the LLIN arm, unlike the other intervention arms, might also be associated with its biting, resting and exophilic behaviours. *Anopheles arabiensis* has marked peak biting activities occurring during early parts of the night well before most people retire to bed [14, 31]. Early evening and outdoor biting of this vector could compromise the efficacy of LLINs, which has been reported as a key challenge that impact entomological outcomes associated with LLINs interventions in Ethiopia [31] and elsewhere in Africa [32]. Likewise, exophilic behaviour of the vector due to exposure to the carbamate insecticide in the IRS intervention arms [26] might contribute to the reduction of the vector densities and HBR, unlike in the LLIN arm. Unfortunately, in this study, there was no assessment of the exophilic rate of the vector; this warrants further research. Based on these findings, it can be suggested that high provision of LLINs alone is not sufficient to control *An. arabiensis* and necessitate complementary interventions.

Results from the outdoor collections by PIT and HLC supported the indoor findings. The IRS + LLIN had a stronger effect on ORD and HBR than the LLIN alone. This might be attributed to more mass killing impact of the carbamate insecticide [26] and more area-wide coverage of the IRS + LLINs interventions versus LLINs alone as explained earlier. However, ORD in the control arm was similar to the IRS arm, but significantly lower than the LLIN arm. Similar and/or lower ORD of *An. arabiensis* in the control arm compared to the intervention arm might be due to indoor resting in the control arm and the influence of a physical barrier provided by LLINs and an exophilic impact of IRS, leading to mosquitoes flying out to feed and rest outdoors. This may add increasing numbers of mosquitoes to the vector population that anyway freely feed and rest outdoors. The impact of IRS + LLIN versus IRS alone on mean ORD and outdoor HBR of the vector were similar. This can be explained in terms of potentially more area-wide coverage, mass killing and exophillic impact of IRS as explained above regardless of the collection venues. It should be noted that outdoor HLC and PIT estimate different entomological parameters and mosquito behaviour.

Furthermore, results showed that none of the mosquitoes tested by ELISA was positive for *P. falciparum* or *P. vivax* circumsporozoite protein, a finding similar to the pre-intervention results from the study area [13] and earlier reports from the district [4, 33]. Despite negative sporozoite ELISA results, there was active malaria transmission taking place during the intervention period [34]. These implicate the need for more sensitive and specialized equipment and techniques such as real time PCR for detection of sporozoite-infected mosquitoes.

The present results are in line with the recent trial in Tanzania which support that combining IRS and LLINs have significant added impact on reducing malaria vector density as compared to LLINs alone [9, 10]. However, the Tanzanian trial targeted both *An. gambiae* s.s. and *An. arabiensis.* They found a significantly lower density and EIR of *An. gambiae* s.s. in IRS + ITN arm compared to the LLIN arm. For *An. arabiensis* there was no density differences between the two arms, but EIR of this species was higher in the LLIN arm than in the combination arm [9]. Both the previous and present trials used LTC for determination of the mosquito densities and sporozoite ELISA for detection of infectivity rates of the vector for malaria.

On the other hand, the present results contrast the recent two trials in Benin [7] and The Gambia [8] that found no significant differences in the density of vector mosquitoes captured by LTC in the IRS + LLIN versus LLIN alone. The reasons for these contrasting results could be explained in terms of differences in vector behaviour and insecticides used for interventions. The Benin trial used bendiocarb (carbamate) and targeted *An. gambiae* s.s. and *Anopheles funestus* while The Gambia trial used DDT and targeted *An. gambiae* s.l. whereas the present study used propoxur (carbamate) and targeted *An. arabiensis. Anopheles gambiae* s.s. is an anthropophagic and endophagic vector, thereby being more susceptible to LLINs compared to the partially zoophagic and exophagic *An. arabiensis*, which is less likely to be affected by LLINs. Okumu et al. [35] suggested that the intervention impact of combining IRS and LLINs is affected by the type of insecticide used. Further potential reason could be due to some level of resistance in local vector populations to the insecticide used on nets and/or spray [36]. In line with argument, it was found that *An. arabiensis* was susceptible to propoxur, insecticide used for spray, but resistant to deltamethrin which is used in LLINs in this study [13].

## Conclusions

Despite using different collection methods targeting host-seeking and resting mosquitoes in both outdoor and indoor settings, there were more mosquitoes found in the absence of interventions and as long as there was an IRS intervention either alone or in combination with LLIN, densities and human biting rates of *An. arabiensis* were the lowest. Moreover, added impact of the combination intervention against malaria infectivity rates of *An. arabiensis* compared to either intervention alone remains unknown and warrants further research.

## Data Availability

The data sets generated and/or analysed during the current study are available from the corresponding author on reasonable request.

## Abbreviations

HSD: host-seeking density
IRD: indoor resting density
ORD: outdoor resting density
HBR: human biting rate
HLC: human landing catch
LTC: light trap catch
IRS: indoor residual spraying
LLINs: long-lasting insecticidal nets
PSC: pyrethrum spray catches
PIT: outdoor artificial pit shelter
